# Cleavage-Independent HIV-1 Trimers From CHO Cell Lines Elicit Robust Autologous Tier 2 Neutralizing Antibodies

**DOI:** 10.3389/fimmu.2018.01116

**Published:** 2018-05-24

**Authors:** Shridhar Bale, Alexandra Martiné, Richard Wilson, Anna-Janina Behrens, Valérie Le Fourn, Natalia de Val, Shailendra K. Sharma, Karen Tran, Jonathan L. Torres, Pierre-Alain Girod, Andrew B. Ward, Max Crispin, Richard T. Wyatt

**Affiliations:** ^1^Department of Immunology and Microbiology, The Scripps Research Institute, La Jolla, CA, United States; ^2^Selexis SA, Geneva, Switzerland; ^3^IAVI Neutralizing Antibody Center, The Scripps Research Institute, La Jolla, CA, United States; ^4^Department of Biochemistry, Oxford Glycobiology Institute, University of Oxford, Oxford, United Kingdom; ^5^Department of Integrative Structural and Computational Biology, The Scripps Research Institute, La Jolla, CA, United States; ^6^Center for HIV/AIDS Vaccine Immunology and Immunogen Discovery, The Scripps Research Institute, La Jolla, CA, United States; ^7^Centre for Biological Sciences, Institute of Life Sciences, University of Southampton, Southampton, United Kingdom

**Keywords:** HIV-1, immunogenicity, viral fusion proteins, vaccines, recombinant protein expression, adjuvants, immunologic

## Abstract

Native flexibly linked (NFL) HIV-1 envelope glycoprotein (Env) trimers are cleavage-independent and display a native-like, well-folded conformation that preferentially displays broadly neutralizing determinants. The NFL platform simplifies large-scale production of Env by eliminating the need to co-transfect the precursor-cleaving protease, furin that is required by the cleavage-dependent SOSIP trimers. Here, we report the development of a CHO-M cell line that expressed BG505 NFL trimers at a high level of homogeneity and yields of ~1.8 g/l. BG505 NFL trimers purified by single-step lectin-affinity chromatography displayed a native-like closed structure, efficient recognition by trimer-preferring bNAbs, no recognition by non-neutralizing CD4 binding site-directed and V3-directed antibodies, long-term stability, and proper N-glycan processing. Following negative-selection, formulation in ISCOMATRIX adjuvant and inoculation into rabbits, the trimers rapidly elicited potent autologous tier 2 neutralizing antibodies. These antibodies targeted the N-glycan “hole” naturally present on the BG505 Env proximal to residues at positions 230, 241, and 289. The BG505 NFL trimers that did not expose V3 *in vitro*, elicited low-to-no tier 1 virus neutralization *in vivo*, indicating that they remained intact during the immunization process, not exposing V3. In addition, BG505 NFL and BG505 SOSIP trimers expressed from 293F cells, when formulated in Adjuplex adjuvant, elicited equivalent BG505 tier 2 autologous neutralizing titers. These titers were lower in potency when compared to the titers elicited by CHO-M cell derived trimers. In addition, increased neutralization of tier 1 viruses was detected. Taken together, these data indicate that both adjuvant and cell-type expression can affect the elicitation of tier 2 and tier 1 neutralizing responses *in vivo*.

## Introduction

Class I viral fusion proteins require cleavage of their single-chain precursors to a meta stable pre-fusion state. The pre-fusion complexes then undergo a series of conformational changes by specific cellular triggers to achieve fusion of host-to-viral membranes, mediating entry (1, 2). These viral surface glycoproteins are also candidates as subunit vaccines. Neutralizing antibodies against functional and conserved epitopes present on several viral glycoproteins protect against acquisition of infection in numerous challenge studies (3–9). Recent development of soluble, native-like mimics of the HIV-1 envelope glycoproteins (Env) as SOSIP and native flexibly linked (NFL) designs stabilize the pre-fusion form of the trimeric protein in large part due to a HR1 gp41 I559P substitution, permitting laboratory-scale production of these trimers (10, 11). The SOSIP variant contains key mutations that enhance cleavage of the Env precursor and tether the resulting gp120 and gp41 subunits *via* a covalent disulfide linkage (10). The NFL variant is a “single-chain,” cleavage-independent design that contains a flexible glycine/serine linker in place of the cleavage site. This modification allows for the proper folding, glycan processing, and quaternary assembly of the trimer (11). Additional stabilizing substitutions in both trimer-types by several investigators result in laboratory-scale trimer production from multiple clades (12, 13).

Both high-resolution crystal and cryo-electron microscopy (EM) structures of the SOSIP trimers reveal a compact, threefold symmetric, native-like conformation as does the cryo-EM reconstruction of the JR-FL native ectodomain (14–20). Similarly, crystal structures of the 16055 and BG505 NFL trimers confirm their native-like state, revealing a quaternary conformation that presents broadly neutralizing epitopes and occludes non-neutralizing determinants (12, 21). The SOSIP trimers have been analyzed in various animal models and elicit autologous tier 2 neutralization in rabbits, non-human primates (NHPs), and heterologous neutralizing antibodies in cows (22–26). Additional stabilizing mutations have been introduced into SOSIP trimers to limit the exposure of V3 to suppress elicitation of non-neutralizing antibodies, to better focus the immune response toward broadly neutralizing determinants (22, 27, 28). The NFL trimers also achieve tier 2 autologous neutralization when immunized into guinea pigs, rabbits, and NHPs (29–32). A recent comparative study indicated that both BG505 SOSIP and BG505 NFL trimers induced roughly equivalent neutralizing antibody responses in NHPs following three immunizations. However, early responses in the NFL-immunized animals were lower than the SOISP-immunized animals (25) and might be attributed to differences in purification or the presence of a His-tag in the NFL trimers.

Despite the capacity to achieve tier 2 neutralization following NFL trimer immunization in guinea pigs, rabbits, and macaques, challenges remain for the development of clinical-grade material for pre-clinical and clinical immunogenicity studies. Laboratory-scale production of NFL (or SOSIP) trimers is typically limited to <3 mg of protein per liter of transiently transfected producer cells (11, 33). Aggregated and disordered trimers, dimeric, and monomeric Env off-target conformations can decrease the yield of well-ordered trimers. Purification by positive selection for SOSIPs and negative selection for NFLs results in homogenous well-ordered trimers suitable for vaccination studies (23, 28, 30, 32–34). Practical issues associated with large-scale current Good Manufucturing Practice (cGMP) production of SOSIP trimers are beginning to be addressed in a recently published study (35). This analysis resulted in a 17 mg/l final well-ordered trimer yield from a total trimer expression of ~114 mg/l using a clinical-grade process and a stable, well-characterized CHO producer line.

Here, we describe in detail the development of a stable research grade CHO-M cell line that produces BG505 NFL trimers with a higher yield of ~1.8 g/l. We characterized the trimers for overall structure, antigenicity, and N-glycan profile. We also evaluated trimers expressed from 293F cells. We demonstrate that the homogeneous BG505 NFL trimers expressed from the CHO-M cell line, when purified to a high level of conformational integrity by negative selection, do not expose V3. The NFL trimers elicit early, robust, and uniform tier 2 neutralizing antibodies in rabbits and remarkably elicit very low V3-directed antibody responses *in vivo*, resulting in neutralization of tier 1 viruses following only the fourth inoculation in adjuvant. Mapping indicated that the elicited tier 2 neutralizing response primarily targets the “N-glycan holes” proximal to residues at positions 241, 230, and 289, as reported previously following immunization of rabbits with BG505 SOSIP trimers (36). These data indicate that if BG505 NFL timers do not expose the V3 region *in vitro*, this immunogenic region remains largely inaccessible *in vivo* following the sensitive process of repeated immunizations in adjuvant. These data have implications for trimer stability designs to “tack down” this region, which are largely unnecessary for well-ordered NFL trimers expressed from a stable CHO cell line.

## Materials and Methods

### BG505 NFL Expression Vector Construction

The gene sequence encoding the BG505 NFL was cloned into the SURE*tech* vector (Selexis, USA). The SURE*technology*™ gene system was used to express the NFL trimers in CHO-M cells. *Env* gene expression was under the control of the EF1 alpha promoter. The SURE*technology*™ expression vectors bear unique genetic elements (called SGEs) that shield the transgene from the silencing effects of surrounding chromatin (37). Transcription is maintained at a maximum level and is independent of the transgene integration site, resulting in stable and high-level protein expression.

### SURE*technology*™ Cell Line Development Platform

The Selexis CHO-M host cell line is derived from CHO-K1 CCL-61 cells from the American Type Culture Collection and has been adapted to grow in suspension in the chemically defined BalanCD Growth A culture medium (Irvine Scientific). Cells were transfected by electroporation using the Neon transfection system (Invitrogen).

### SURE *CHO-Mplus Libraries*™ Technology

To support the proper folding and secretion of the complex and difficult-to-express recombinant HIV-1 Env trimer, Selexis developed CHO-M*plus* libraries. This technology is based on the co-expression of assistant proteins involved in the folding, assembly or trafficking cellular machineries and, as well, general cell metabolism (38). We applied the CHO-M*plus* libraries approach to BG505 NFL expressing CHO-M cells to support proper folding and assembly of well-ordered trimeric HIV spikes to further improve secretion and yield.

### Single-Cell Cloning Using ClonePix FL Device

The same medium (BalanCD Growth A, Irvine Scientific) was used as a basal medium for transfection, single-cell cloning, and production in order to keep the environment of the cells unchanged throughout the whole procedure. Following transfection of BG505 NFL containing SURE*tech* vector, we applied hygromycin selection pressure to generate the stable pools. Diluted cells were plated into semi-solid media (CloneMedia©; Molecular Devices) and plates were incubated at 37°C with 5% CO_2_, in a humidified incubator. Expanded colonies were picked using ClonePix™ FL Imager from Molecular Devices and transferred to 96-well plates, then expanded in first 24-well and then 6-well TC plates.

### Fed-Batch Performance Evaluation

Growth and production performance of individual clones were evaluated in 125 ml shake flasks to select the best clones by the criteria of cell productivity, cell line stability and BG505 NFL trimer production. The best performing clones were expanded to 500 ml in a 10-day fed-batch process for trimer production using Acti CHO A+B feed (GE Healthcare, USA). Fed-batch cultures were initiated at cell concentrations of 0.3 × 10^6^ cells/ml.

### ELISA, Trimer Purification, and Antigenicity Assays

To assess the yield and quality of the well-ordered BG505 NFL trimers, fed-batch production supernatants were analyzed by ELISA and blue-native PAGE. Trimer production was evaluated using ELISA plates coated with anti-His mAbs to capture NFL trimers, followed by the incubation with the trimer-specific bNAb, PGT145. Plates were incubated with peroxidase-conjugated goat anti-human secondary antibody and developed using 3,3′,5,5′-Tetramethylbenzidine. To confirm production yield, crude supernatants were resolved by 4–12% Bis-Tris SDS-PAGE analysis (NuPAGE, Invitrogen).

Culture supernatants were subsequently purified on *Galanthus nivalis*-agarose column, and Superdex 200 size-exclusion chromatography (SEC) column before analysis by BN-PAGE. For IP-based antigenicity assays, a panel of mAbs was used: the CD4bs-directed bNAb VRC01; the trimer-preferring bNAbs VRC06, PGT145, and PG9; and the non-neutralizing, CD4bs-directed mAb, F105. Briefly, supernatant was incubated with each mAb and BG505 NFL+ mAb complexes were captured on the solid phase from solution using protein-A agarose beads. Following extensive PBS washing, eluates in boiling SDS reducing gel sample buffer were loaded onto gels to analyze levels of relative binding.

For immunization and N-glycan studies, supernatants from pool Z were used to isolate the well-ordered BG505 NFL trimers. After lectin chromatography and SEC, the trimers were purified to homogeneity by negative selection using the non-neutralizing mAb, F105, in complex with protein A resin. An additional second SEC step was used to select exclusively for well-ordered and highly homogenous BG505 NFL trimers.

### EM and Data Processing

BG505 NFL trimers were applied to glow-discharged carbon coated mesh grids (400-Cu) for 15 s and stained with 2% uranyl formate for 30 s. The grids were analyzed using a FEI Tecnai Spirit Electron Microscope operating at 120 kV using an electron dose of ~30 e^−^/Å^2^. Images were collected using a Tietz 4k × 4k TemCam-F416 CMOS camera. Data were uploaded into the Appion database and particles were selected using DoG, two dimensional class averages were obtained using iterative multivariate statistical analysis/multireference alignment (39, 40). The class averages were examined visually to determine the quality of the trimers (closed, open, and non-native like trimers) as described previously (11, 41).

### N-Glycosylation Analysis by Hydrophilic Interaction Ultra-Performance Liquid Chromatography (HILIC-UPLC)

N-linked glycans were enzymatically released from the purified BG505 NFL trimers using Peptide-N-Glycosidase F (PNGase F). Glycans were then fluorescently labeled with 2-aminobenzoic acid and analyzed by HILIC-UPLC, as described previously (22, 42, 43). The abundance of oligomannose-type glycans was determined by digestion of released glycans following digestion with EndoH (22).

### Site-Specific N-Glycosylation Analysis

A sample-specific glycan library was created by ion mobility mass spectrometry of the total pool of PNGase F-released glycans, as previously described (43). Glycoproteins were reduced, alkylated, protease digested (trypsin or chymotrypsin), and enriched for glycopeptides, as described before (43, 44). Enriched glycopeptides were analyzed by liquid chromatography–electrospray ionization-tandem mass spectrometry on a Q-Exactive Orbitrap mass spectrometer (Thermo Fisher Scientific), as previously described (43). Analysis of the data was performed using Byonic™ (Version 2.7) and Byologic™ software (Version 2.3; Protein Metrics Inc.) (43).

### Immunization Experiments

Immunization experiments with BG505 NFL from CHO-M cell lines were performed at Covance (Denver, PA, USA) and experiments with BG505 NFL/SOSIP from 293F cells were performed at ProSci Inc. (Poway, CA, USA). New Zealand white female rabbits were inoculated subcutaneously with 30 µg of BG505 NFL or BG505 SOSIP trimers at two sites. The BG505 NFL from CHO-M cells were formulated with 75 U of ISCOMATRIX (CSL) adjuvant and the BG505 NFL and BG505 SOSIP from 293F cells were formulated with 10% Adjuplex (v/v) (Advanced BioAdjuvants LLC.; Sigma) immediately prior to immunization. Inoculations were performed at 0, 4, 12, and 24 weeks and whole blood was collected 14 days after each inoculation and, as well, in between the repeated immunizations. The BG505 NFL/SOSIP-immunized animals at ProSci Inc. received an additional Env boost at week 32. Two animals received PBS with adjuvant as a control for each of the immunization studies. In addition, pre-bleeds were collected for all animals prior to the immunization regimen.

### ELISA Analysis

BG505 NFL/SOSIP-specific IgG-binding titers were determined using ELISA. The 96 half-well ELISA plates (Corning Incorporated) were coated with anti-His antibody (mouse) and BG505 NFL- or BG505 SOSIP-His-tagged trimers, at concentration of 2 µg/ml, were captured overnight at 4°C. The plates were incubated for 1 h with a blocking buffer comprising 2% non-fat milk in PBS + 5% fetal bovine serum. The plates were washed with PBS + 0.2% Tween-20 and incubated for 1 h with fivefold serial dilutions of rabbit sera or IgGs at a starting concentration of 10 µg/ml. Plates were washed with PBS + 0.2% Tween-20 and incubated with horseradish peroxidase coupled anti-rabbit/anti-human IgG at 1:5,000 dilution for 1 h and developed with HRP-TMB substrate solution. The HRP-TMB reaction was stopped with 0.3 N sulfuric acid and absorbance was measured at 450 nm. Binding data were analyzed using GraphPad Prism.

### Serum Neutralization Analysis

The TZM-bl assay, as described previously, was used to determine the level of serum neutralization (45). Serum samples were heat-inactivated at 56°C for 45 min prior to neutralization analysis. Serial dilutions of serum were incubated with HIV-1 Env pseudovirus for 1 h and TZM-bl cells were added to the mixture. Relative light units based on luciferase activity were measured following 2 days of serum-virus-target cell incubation. Data were analyzed using GraphPad Prism and the reciprocal serum dilution at which 50% of virus neutralization was achieved (ID_50_) is reported.

## Results

### Generation of CHO-M Clonal Cell Lines Expressing BG505 NFL Trimers

**Figure 1:**
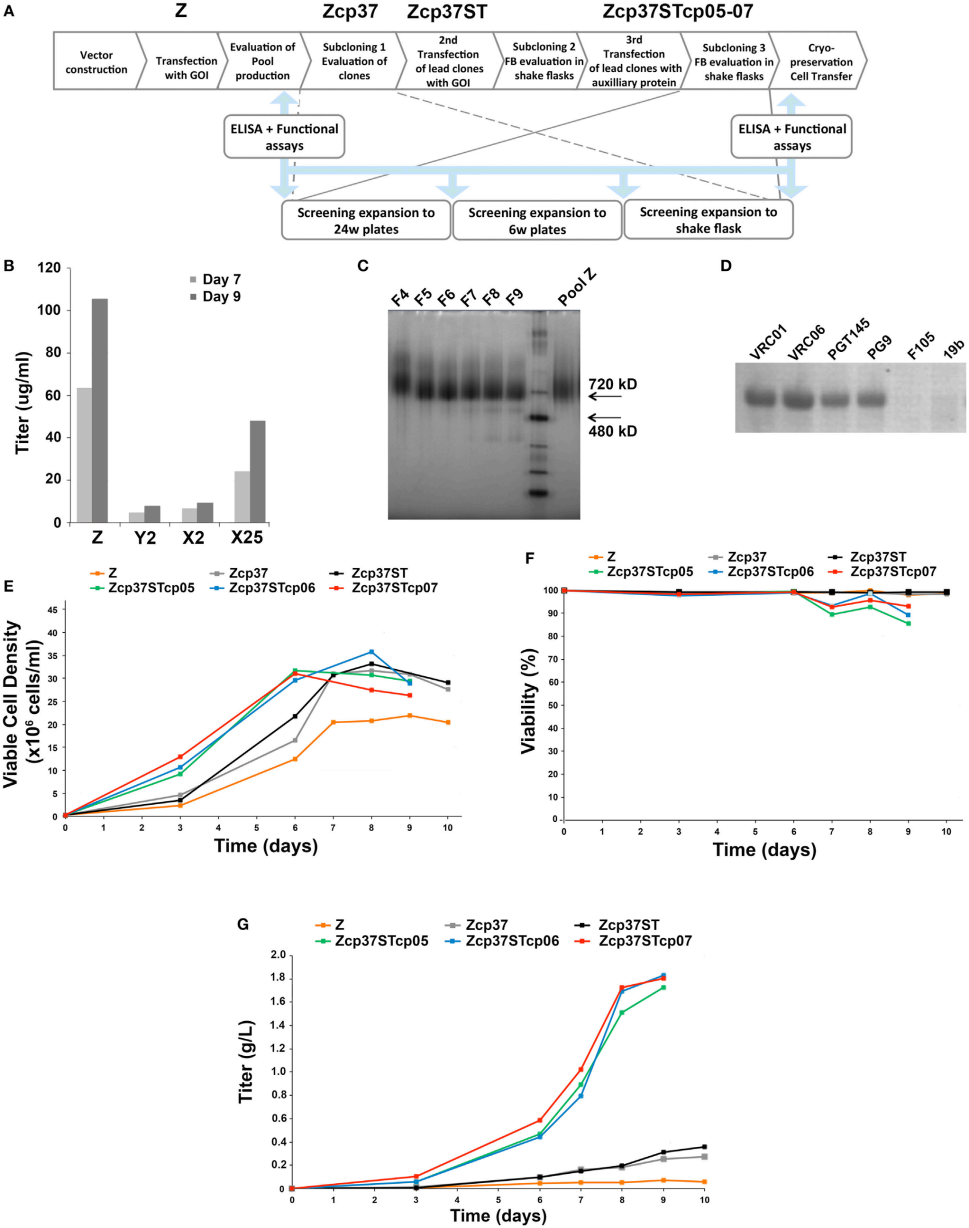
Growth and production performance of BG505 NFL expressing CHO-M pools and subclones. **(A)** Schematic of Selexis *SUREtechnology*™ cell line development procedure. The pool/clones identified and developed at different stages of trimer production are labeled on top of the schematic. **(B)** Four different CHO-M pools were generated and cultivated in fed-batch conditions. BG505 NFL production (day 7, day 9) was analyzed by ELISA binding. Pool Z was selected for further development. **(C)** BG505 NFL trimers produced by pool Z was purified *via* GNL resin and resolved by size-exclusion chromatography. Collected fractions (F4–F9) were analyzed by BN-PAGE. **(D)** Supernatants of pool Z were immunoprecipitated using selected antibodies. Efficient recognition was detected for trimer specific/preferring bNAbs (VRC01, VRC06, PGT145, and PG9) and negligible binding for non-bNAbs (F105 and 19b). **(E)** Growth and **(F)** viability plots of CHO-M clones isolated from pool Z and cultured in fed-batch. **(G)** BG505 NFL production in various CHO-M cell lines as assessed by ELISA titers. The best performing clones (Zcp37STcp05-07) produced five-fold higher level of trimer when compared to the initial clones, Zcp37 and Zcp37ST.

We used Selexis SURE*technology*™ cell line development platform to generate CHO-M cells lines that produced the BG505 NFL trimers (schematic, Figure 1A). Following transfection of plasmid DNA and antibiotic selection, four stable CHO-M pools (pools Z, Y2, X2, and X25) were identified and evaluated for Env trimer production using PGT145 ELISA binding analysis. Among the CHO-M pools generated, pool Z demonstrated the best level of expression with yields >2-fold higher than the next best performing pool (Figure 1B). To assess the quality of trimers produced, cell culture supernatant derived from pool Z was purified using a lectin-affinity column (GNL-agarose), followed by a size-exclusion column (SEC; S200, GE Healthcare). Following SEC, blue-native PAGE analysis of the column fractions revealed high levels of trimer with minor levels of aggregates (Figure 1C). To assess the antigenicity of CHO-M expressed BG505 NFL trimers, immunoprecipitations (IPs) were performed on the supernatant from pool Z. BG505 NFL trimers from the supernatant were efficiently immunoprecipitated by the trimer-preferring bNAbs VRC06, PGT145, and PG9, resulting in a strong trimer bands on SDS gels. By contrast, the non-neutralizing antibodies F105 and 19b resulted in weak trimer bands (Figure 1D). These data indicate that the cells in pool Z expressed a very high proportion of native like well-folded BG505 NFL trimers. These analyses likely detect trimers from the predominant, most highly expressing clone in the pool Z cell population.

We subsequently used cells from pool Z to isolate individual clones expressing the BG505 NFL trimers. Clones in fed-batch cultivation were evaluated for growth and trimer production. Among the individual clones isolated, Zcp37 demonstrated the best parameters in term of cell growth, viability, trimer production, and trimer quality. The Zcp37 clone was transfected a second time to obtain the Zcp37ST cell pools, further improving trimer production. Growth, viability, and Env titers derived from pool Z, clone Zcp37, and pool Zcp37ST were sampled daily and are shown in Figures 1E,F, respectively. ELISA binding analysis confirmed a ~3-fold increase of trimer production from the original pool Z to the subclones, Zcp37 and Zcp37ST (Figure 1F). The pool Zcp37ST resulted in a yield of ~400 mg/l and this oligo-pool of cells was selected for further development aimed at increasing trimer yield.

The generation of CHO-M cell lines with increased trimer production is highly challenging for the cell and warrants addressing expression and secretory issues. For example, overloading the secretory pathway can cause oxidative stress, metabolic unbalance, or early apoptosis during cell line establishment or manufacturing development. These unfavorable events are especially true for difficult-to-express proteins and co-expression of auxiliary molecular partners for proper assembly, maturation, or if activation of the target protein is necessary (38, 46). To address this issue, we applied the CHO-M*plus* libraries approach by transfecting the Zcp37ST pool with auxiliary proteins to support proper trimer assembly and to further improve trimer yield. After transfection, selection, and final subcloning, three different clones Zcp37STcp05, Zcp37STcp06, and Zcp37STcp07, co-expressing assistant proteins, were evaluated in fed-batch cultivation for growth and production performance of the NFL trimers. Cultures were sampled daily for growth, viability, and titer (Figures 1E–G). Compared to the initial clones, a ~6-fold increase in trimer production was detected with the new set of subclones, resulting in final production yields of ~1.8 g/l of BG505 NFL trimer for all the three subclones (Figure 1G).

### Characterization of BG505 NFL Trimers Expressed in CHO-M Cell Lines

**Figure 2:**
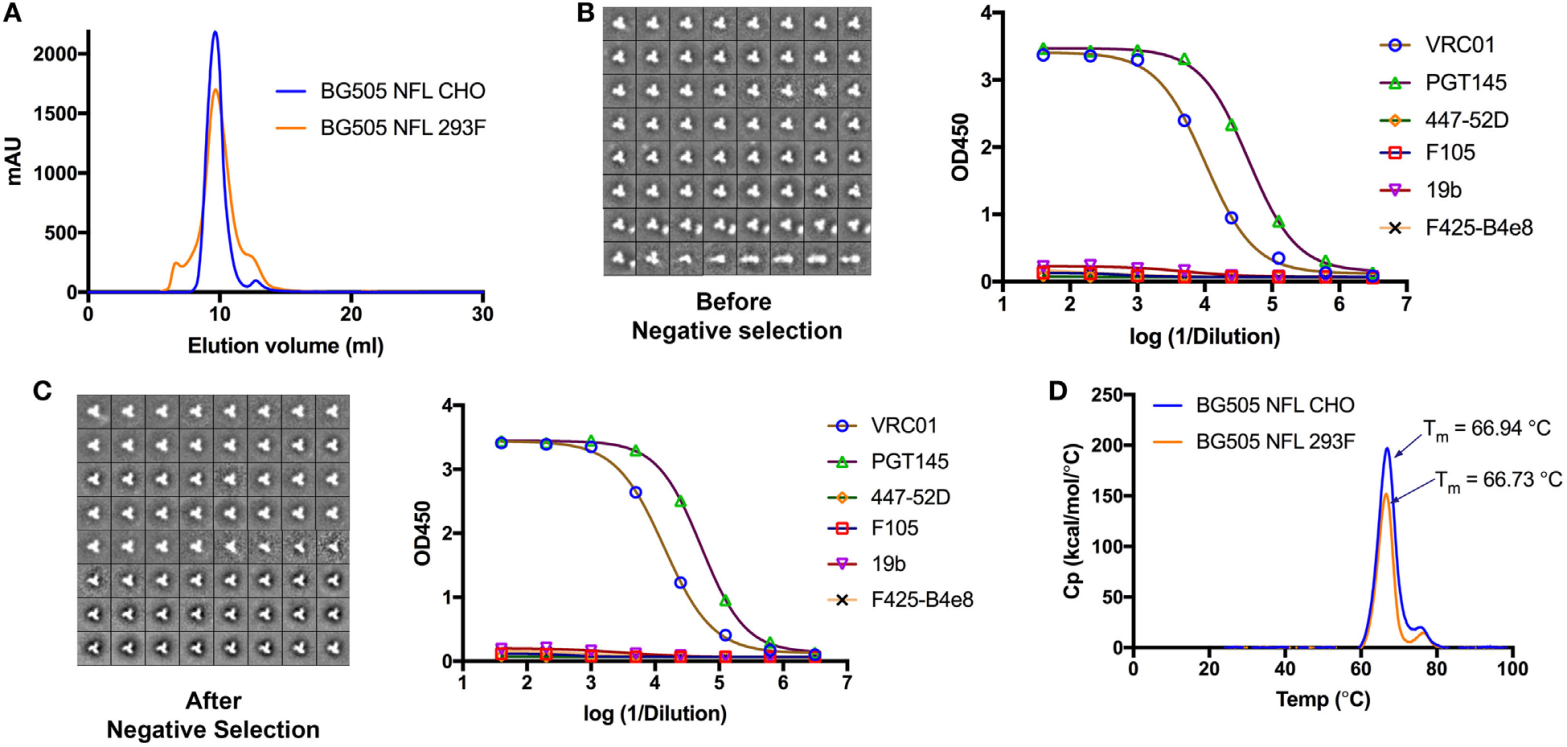
Characterization of CHO-M cell-produced BG505 NFL trimers. **(A)** We superimposed size-exclusion chromatography (SEC) profiles of BG505 NFL produced from CHO-M and 293F cells, respectively, following purification from a lectin-affinity column. The trimers produced from CHO-M cells were more homogenous while the oligomers expressed from the 293F cells displayed predominant trimer and detectable levels of aggregate, dimer, and monomer fractions. **(B)** Negative stain electron microscopy (nsEM) and ELISA binding analysis of BG505 NFL trimers produced in CHO-M cells from the peak fraction following lectin-affinity and SEC isolation. **(C)** nsEM and ELISA analysis of BG505 NFL trimers following F105-based negative selection. **(D)** Melting profiles of BG505 NFL trimers produced in CHO-M and 293F cells as measured by differential scanning calorimetry.

All the characterization and analysis of BG505 NFL expressed in the CHO-M cells and shown in Figure 2 were performed on a single batch of supernatant from an initial pool Z. BG505 NFL trimers in the supernatant of pool Z were purified over a lectin-affinity column and further purified by SEC (S200, GE Healthcare) as described previously (11). The trimers resolved by SEC were detected by UV absorbance at 280 nm as a major single peak containing mostly trimers and a minor peak comprised of smaller oligomers. The major peak resulted in a yield of ~100 mg of trimer from 1 l of supernatant. A superposition of the elution profiles of the BG505 NFL trimers expressed in CHO-M cells compared to expression in 293F cells revealed that the latter contained larger fractions of aggregated trimers, dimeric, and monomeric components (Figure 2A). The trimers from the peak fraction from pool Z were further examined using negative-stain electron microscopy (nsEM) to reveal a composition of 90% closed native-like trimers and 10% non-native trimers (Figure 2B). The trimers also displayed favorable antigenic properties as assessed by binding analysis with bNAbs and non-bNAbs by ELISA. BG505 NFL trimers were efficiently recognized by the bNAbs PGT145, VRC01, while negligible recognition was detected by the non-broadly neutralizing mAbs F105 and the V3-specific NAbs 447-52D, 19b, and F425-B4e8 (Figure 2B). A negative selection step was implemented to separate the small fraction of non-native like trimers from the well-ordered trimers (47). Following negative selection, the BG505 NFL trimers were ~97% closed native-like and 3% non-native like as determined by nsEM. The trimers also maintained a favorable antigenic profile, that is efficient recognition by the bNAbs, and minimal recognition by the non-bNAbs, similar to trimer fractions assessed prior to negative selection (Figure 2C). As discussed in the previous section, subsequent subclones isolated from pool Z improved the yield of the trimers with no detectable difference in the quality of the trimers. Characterization of trimers from the final subclones is shown in Figures S1A–C in Supplementary Material. The majority SEC peak from the supernatant of final subclones resulted in a yield of ~410 mg/l of well-ordered trimers.

The stability of BG505 NFL trimers from CHO-M cells were determined under two conditions: (1) rapid five-time freeze:thaw cycling and (2) storage for 2 months at 4°C. For the freeze thaw analysis, the trimers were flash frozen in liquid nitrogen and then thawed immediately on a heat bath at 37°C and the process was repeated for five times. No variation was observed in the antigenic properties of the trimers after the rapid free thaw cycle or long-term storage (Figure S1D in Supplementary Material). We further examined the thermostability of the BG505 NFL trimers expressed in both CHO-M and 293F cell lines by differential scanning calorimetry (DSC). BG505 NFL trimers expressed in CHO-M cells displayed a single-peak T_m_ at 66.94°C, indicating trimer homogeneity. For comparison, trimers expressed in 293F cells melted at a comparable T_m_ of 66.73°C (Figure 2D).

**Figure 3:**
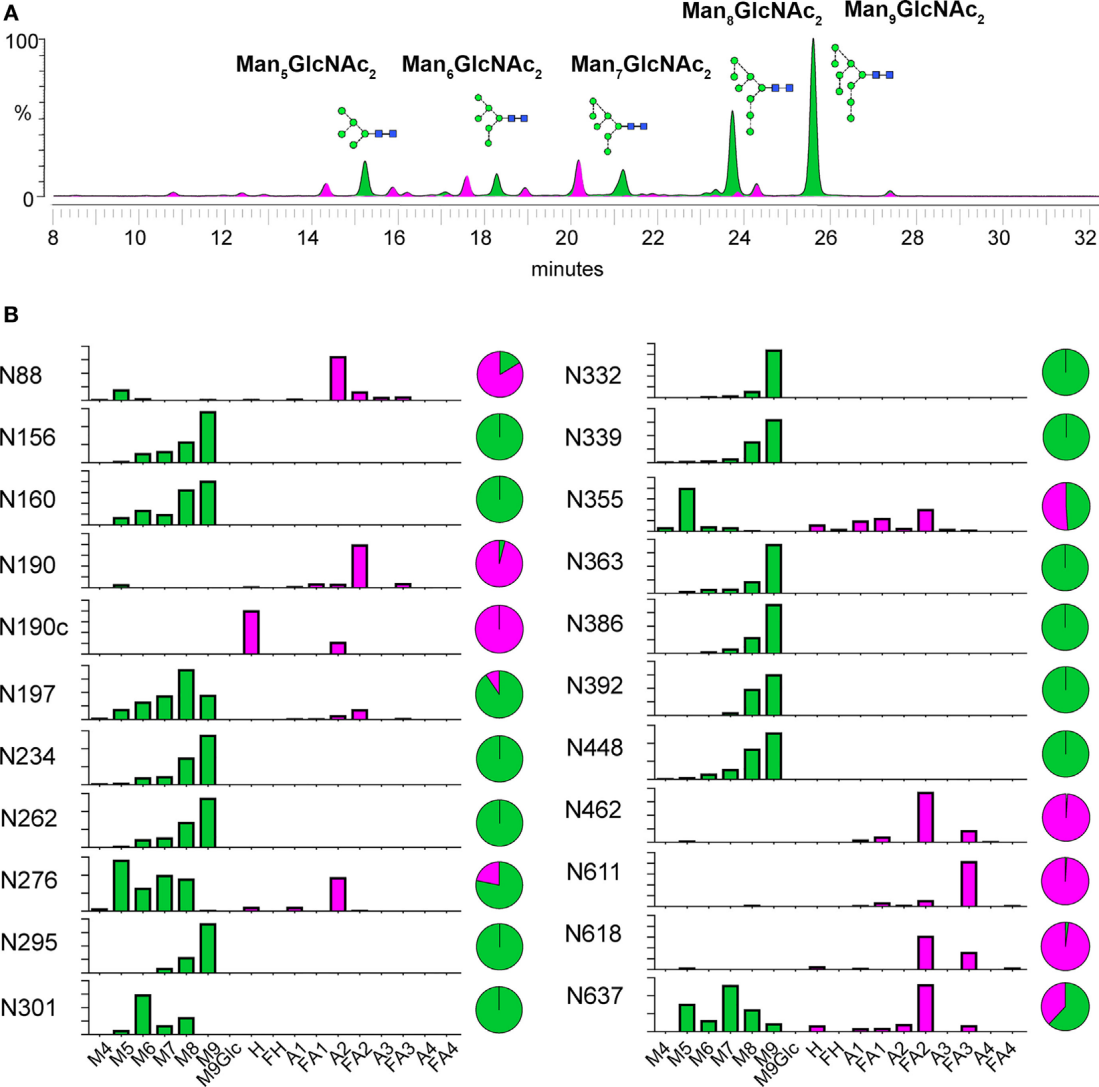
Glycosylation analysis of BG505 NFL trimers produced in CHO-M cells. **(A)** Overall glycan composition. Hydrophilic interaction ultra-performance liquid chromatography spectra of fluorescently labeled N-linked glycans released from BG505 NFL produced in CHO-M cells. Oligomannose-type and hybrid-type glycans are colored green and complex-type glycans are colored pink. The corresponding structure for peaks of oligomannose glycans (Man_5–9_GlcNAc_2_) are shown and labeled accordingly. **(B)** Site-specific N-glycosylation analysis. Relative quantification of the microheterogeneity of 22 of 28 BG505 NFL N-glycosylation sites is shown. The trimers were protease digested and analyzed by liquid chromatography–electrospray ionization-tandem mass spectrometry. The bar graphs represent the mean of two analytical replicates and the pie charts display the overall abundance of oligomannose-type (green) and complex- and hybrid-type (magenta) glycans. A sample-specific glycan library used as the basis for this analysis is shown in Table S1 in Supplementary Material. Details of glycopeptide peaks identified are shown in Table S2 in Supplementary Material. Identified glycoforms were grouped according to the number of their antennae. The glycan names are as previously described (43) and as follows: Mn = number (n) of mannose residues; An = number (n) of antennae (e.g., A2 = biantennary); Gn = number (n) of galactose residues; H = hybrid residues; F indicates the presence of a core fucose.

We performed overall and site-specific analysis of N-linked glycosylation to analyze the composition of the glycan shield of BG505 NFL trimers in detail (Figure 3). The overall glycan profile of BG505 NFL trimers from CHO-M cells is typically oligomannose-dominated, as reported previously for the native-like SOSIP trimers (22, 44) (Figure 3A). Under-processed glycosylation typically serves as an indicator for native-like trimer folding (22, 44, 48). The N-glycans on the trimers were digested by EndoH and analyzed by HILIC-UPLC, which revealed that the percentage of oligomannose-type glycans for BG505 NFL trimers was 68%. For comparison, similar treatment of BG505 SOSIP trimers results in 60–80% of oligomannose glycans (22, 44, 48). The processing of individual sites also mirrored those reported for the corresponding BG505 SOSIP.664 trimers (Figure 3B) (43, 44).

### BG505 NFL Trimers From CHO-M Cells Elicit Robust Autologous Tier 2 Neutralizing Antibodies

**Figure 4:**
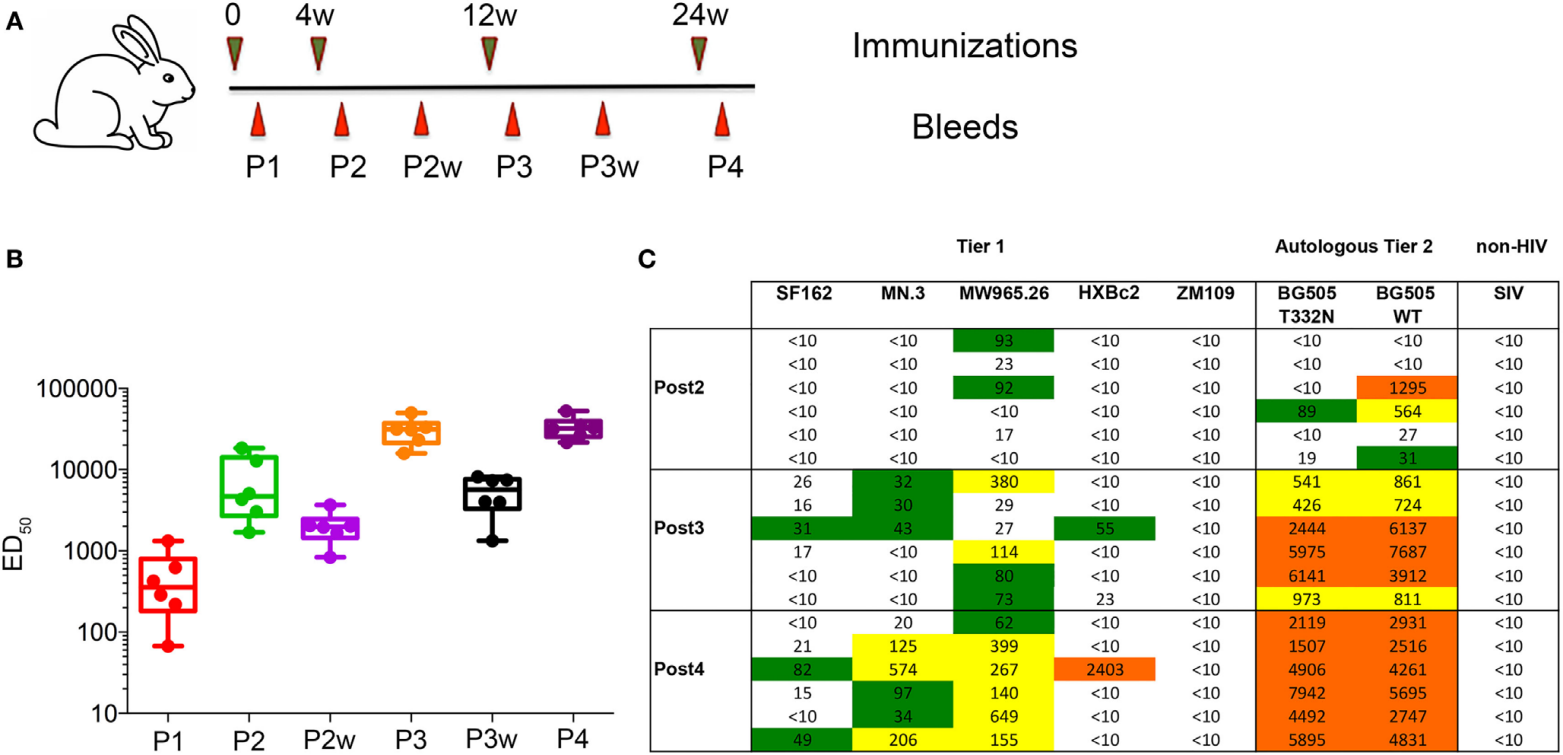
BG505 NFL trimers produced in CHO-M cell lines elicit robust tier 2 neutralizing antibodies. **(A)** Schedule of immunizations and bleeds. **(B)** Longitudinal analysis of BG505 NFL-specific antibodies elicited in rabbits. The geometric mean of binding titer (ED_50_) for all the animals is plotted against various bleed time points. **(C)** Neutralization ID_50_ titers at sampling bleeds following P2, P3, and P4 against a panel of autologous tier 2 and tier 1 viruses as determined by the TZM-bl pseudovirus entry assay. Data are representative of three independent measurements for each virus in the panel.

We immunized six rabbits with 30 µg each of highly homogenous, non-V3-exposing BG505 NFL trimers purified from pool Z and formulated in ISCOMATRIX adjuvant at 0, 4, 12, and 24 weeks, respectively. Serum samples were collected 2 weeks following each immunization and, as well, at intermediate time points between immunizations (Figure 4A). Two rabbits were inoculated with PBS and adjuvant to generate negative control sera. ELISA analysis was performed at each sampling time point to determine Env-specific antibody binding titers. BG505 NFL trimer-specific antibodies were detected 2 weeks following the first immunization in all the rabbits and peaked following three immunizations (Figure 4B). A long rest period of 12 weeks, followed by a fourth immunization, marginally improved the binding titers in selected animals.

The serum samples were further assessed for the capacity to neutralize autologous BG505 tier 2 pseudoviruses and tier 1 viruses by the standard TZM-bl assay (Figure 4C). Tier 2 autologous neutralization was detected in four of six rabbits against wild-type BG505 pseudovirus following only two immunizations (the serum sample at 6 weeks). Because the BG505 NFL trimer immunogen had the N-glycan restored at residue N332, we assessed and detected very weak neutralization in two rabbits against the antigen-matched BG505 T332N pseudovirus. No neutralization was detected against the relatively neutralization-sensitive tier 1 viruses SF162, MN.3, HXBc2, and ZM109. Following two inoculations, two rabbits elicited antibodies that could weakly neutralize the very V3-sensitive MW965.26 pseudovirus.

Following three immunizations, the serum from all the six rabbits robustly neutralized the wild-type tier 2 BG505 autologous virus and the antigen-matched BG505 T332N pseudovirus. By contrast, weak neutralization was detectable in 3–4 rabbits against the tier 1, V3 mAb-sensitive pseudoviruses SF162, MN.3, and MW965.26. The inability to efficiently neutralize the tier 1 viruses likely reflects the maintenance of the native-like and desired closed native trimer conformation during the 12 weeks of the regimen. These results indicate that the BG505 NFL trimers do not appreciably expose the highly immunogenic V3 region at *in vivo* physiologic temperatures, following transport to the lymph nodes (LN)/germinal centers from the site of administration. Two rabbits did elicit antibodies that could weakly neutralize the HXBc2 pseudovirus. These data likely indicate the presence of low levels of tier 1 “F105-like mAbs” as HXBc2 is somewhat mismatched in V3 relative to the BG505 Env.

The fourth immunization further improved neutralization titers against the autologous tier 2 BG505 pseudoviruses. Serum from all the six rabbits neutralized wild-type BG505 pseudovirus and BG505 T332N pseudovirus with equivalent titers that were uniform across all the animals. The geometric mean of neutralization titers (serum dilution factor at which 50% neutralization is achieved, or ID_50_) against wild-type BG505 pseudovirus was 3,653 (geometric SD factor 1.40) and against BG505 T332N pseudovirus was 3,857 (geometric SD factor 1.89). The neutralization of the V3-sensitive tier 1 viruses SF162, MN.3, and MW965.26 was slightly higher following the fourth immunization, likely indicating the elicitation of some V3-directed responses. In addition, neutralization was not observed against ZM109 virus after four immunizations and serum from only one rabbit neutralized HXBc2 pseudovirus (Figure 4C).

Weak neutralization was previously reported in rabbits and NHPs immunized with BG505 SOSIP and V3-loop stabilized variants against a representative HIV global panel of tier 2 viruses (25, 33). The neutralization was also limited to 4–5 viruses from the panel and to a subset of immunized animals, illustrating the challenges in achieving cross-neutralization breadth. Accordingly, we tested if serum from the any of the BG505 NFL trimer-immunized rabbits neutralized heterologous tier 2 viruses. A panel of 11 “in-house” heterologous tier 2 viruses was analyzed. Seven viruses from the panel were neutralized by serum from at least one rabbit with a neutralization titer >30 (Figure S2 in Supplementary Material). Among the pseudoviruses, the best neutralization was achieved against RW020, 398F1, and CNE8 with serum from three or more animals neutralizing these viruses. The specificity of the neutralization detected in the anti-sera of these animals was not determined.

### Rabbits Elicit Neutralizing Antibodies Against “N-Glycan Holes”

**Figure 5:**
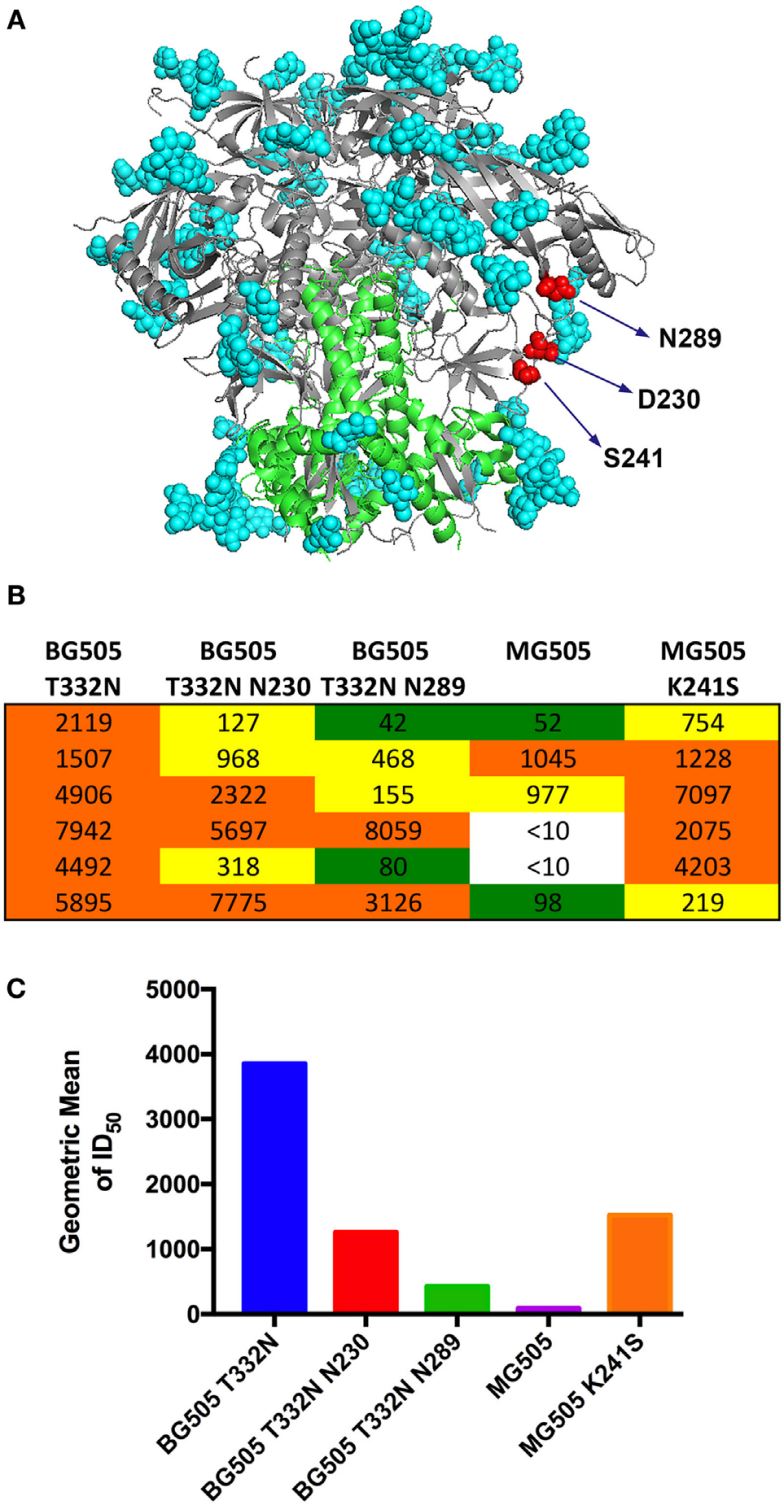
Antibodies elicited by BG505 NFL trimers target holes in the N-glycan shield. **(A)** Cartoon representation of the BG505 SOSIP trimer (PDB id 5CEZ) depicting known N-glycan holes at positions 289, 241, and 230, respectively. The backbone for gp120 is colored gray and gp41 is colored green. The amino acids at the position of the glycan holes are shown as red spheres and ordered glycans as seen in the crystal structure are shown as cyan spheres. **(B)** Neutralization ID_50_ titers at the final bleed point (P4) against a panel of autologous tier 2 BG505 viruses that have the glycans restored at positions 230 and 289 are shown. Elicitation of antibodies against the glycan hole at position 241 was probed against the parent virus MG505 that lacks the glycan at position 241 and the MG505 K241S variant that restores a serine residue at position 241 as it exists in wild-type BG505 virus. **(C)** Geometric mean of neutralization ID_50_ titers showing reduction in neutralization against a panel of pseudoviruses with N-glycans restored.

We mapped the activity in the serum samples to investigate if rabbits immunized with BG505 NFL trimers from CHO-M cells targeted the glycan holes naturally present on the BG505 Env trimers (Figure 5A), as reported following BG505 SOSIP immunization (36). To accomplish this analysis, we performed neutralization assays against two types of pseudoviruses. In the first approach, we compared neutralization of BG505 T332N pseudovirus against pseudoviruses that possess N-glycan PNG motifs restored at positions N289 and N230, respectively. A decrease in neutralization titers against a glycan-restored pseudovirus would indicate the presence of antibodies against the N-glycan hole. Decreased neutralization was observed in three rabbits against BG505 T332N N230 pseudovirus and in four rabbits against BG505 T332N N289 pseudovirus (Figure 5B). In the second approach, we investigated antibodies against glycan hole at residue K241 using the parent MG505 pseudovirus and the MG505 K241S variant, obtained from the Burton laboratory. We chose this alternate approach due to difficulties expressing BG505 pseudovirus with the N-glycan restored at 241, which does not yield infectious virus. Both BG505 and MG505 viruses lack the N-glycan at residue 241, and the amino acid sequence varies at 13 residues between these two related viruses. The MG505 virus (derived from the *M*other) contains a lysine residue at residue 241 while the BG505 virus (derived from the *B*aby) contains a serine residue at that position (36). Decreased neutralization was observed in five rabbits against MG505 pseudovirus, indicating that the antibodies elicited against BG505 NFL do not recognize efficiently the glycan hole at residue 241 in MG505 virus. However, neutralization was restored in all five rabbits when the lysine at position 241 was back-converted to a serine in the MG505 pseudovirus context, which matches the BG505 sequence at this site (Figure 5C). The restoration of neutralizing activity indicates that these rabbits elicit antibodies against the glycan hole located proximal to residue 241 and that these antibodies specifically recognize the serine residue present in this glycan hole.

### BG505 NFL Responses Compared to SOSIP Trimers Inoculated Into Rabbits

**Figure 6:**
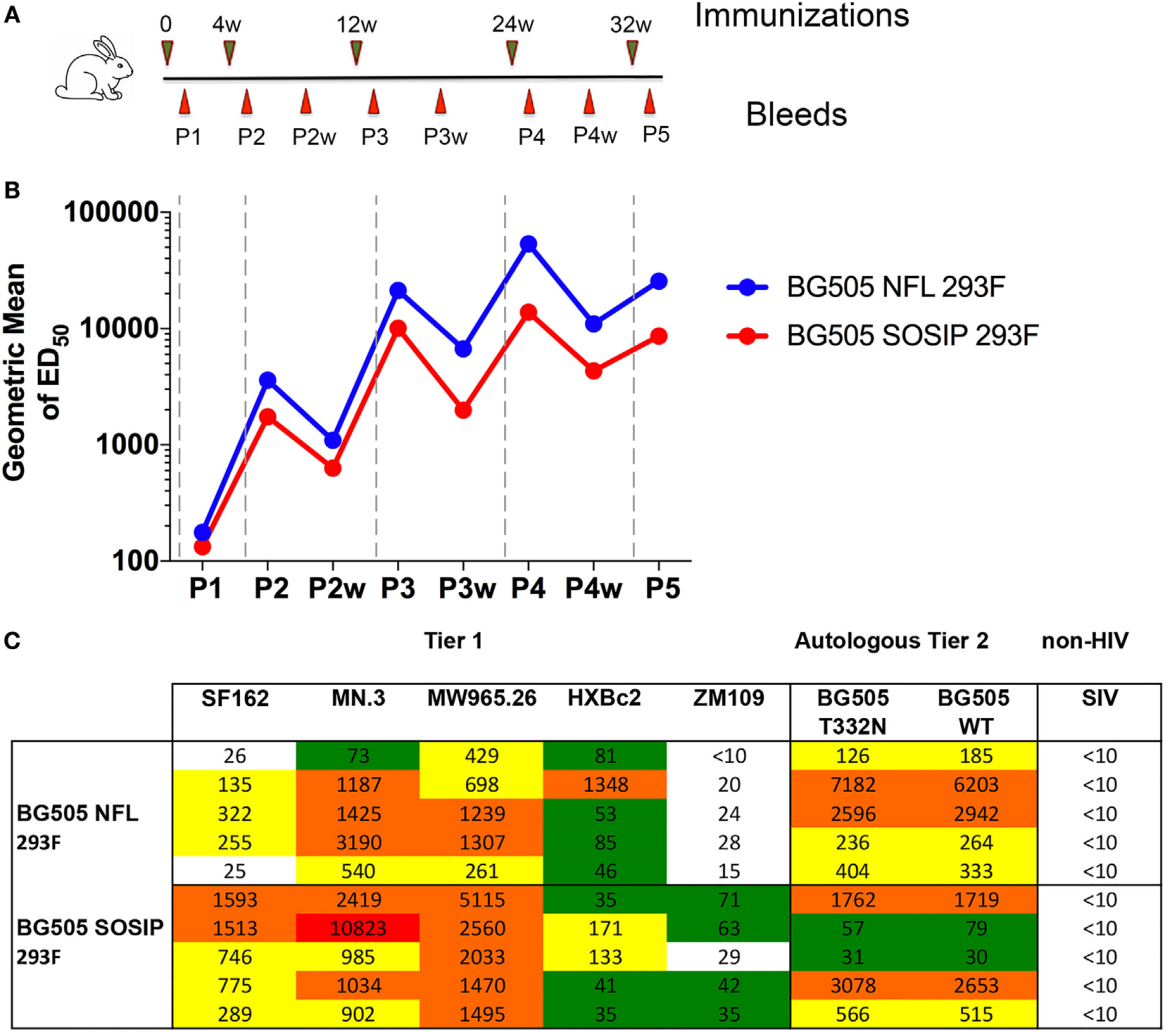
Comparison of neutralizing responses elicited in rabbits by BG505 NFL and BG505 SOSIP trimers. **(A)** Schedule of immunizations and bleeds. **(B)** Longitudinal analysis of BG505 NFL- or BG505 SOSIP-specific antibodies elicited in rabbits. The geometric mean of ELISA binding titers (ED_50_) for all the animals is plotted against selected sampling bleed time points. Immunization events are shown as vertical dashed gray lines. **(C)** Neutralization ID_50_ titers at final bleed point (P5) against a panel of tier 1 and tier 2 pseudoviruses. Data are representative of two independent measurements.

Rabbits were immunized with 30 µg each of BG505 NFL or BG505 SOSIP trimers expressed in 293F cells and purified in similar fashion using F105 affinity column negative selection as described previously (47). Each group was comprised of five animals and a control group of two rabbits received PBS with adjuvant. Immunizations were done at 0, 4, 12, and 24 weeks and the trimers were formulated with Adjuplex to a final composition of 10% (v/v) prior to inoculation. The immunizations and bleeds schedule are shown in Figure 6A. Serum from the rabbits was analyzed for binding titers and neutralization. BG505 NFL- or BG505 SOSIP-specific antibody binding titers were detected in animals from both the NFL and SOSIP groups after two immunizations. Binding titers improved with further immunizations and exhibited the typical “saw tooth” pattern as seen with other experiments involving BG505 NFL trimers (25, 29). The binding titers of the NFL-immunized animals were better than the SOSIP-immunized animals at all the serum bleed points tested (*p* = 0.0831 at P4, *p* = 0.0046 at P5) (Figure 6B). However, the difference in the binding titers did not translate to differences in autologous neutralization titers (Figure 6C). After four immunizations, strong neutralization was achieved by the serum of only two rabbits in each of NFL and SOSIP groups (Figure S3 in Supplementary Material). In addition, weak neutralization was achieved in two other rabbits derived from the NFL trimer-immunized group while all other animals had neutralization titers <30. To improve the neutralization, we immunized the rabbits with an additional boost at 32 weeks. The neutralization titers against BG505 T332N and BG505 wild-type pseudoviruses improved in animals derived from both the groups. The neutralization titers for the NFL-immunized group of animals ranged from 126 to 7,182 with a geometric mean of 741 against the BG505 T332N pseudovirus. The neutralization titers for the SOSIP-immunized animals ranged from 31 to 3,078 with a geometric mean of 352 against the same pseudovirus. In addition, the neutralization titers were not significantly different (*p* = 0.5132), similar to the non-significant differences in BG505 autologous neutralization from the NFLs compared to SOSIPs in NHPs (25). Roughly, equivalent neutralization titers were also observed against wild-type BG505 pseudovirus from samples derived from animals in both groups.

Sera from both the groups also neutralized a panel of tier 1 pseudoviruses tested (Figure S4 in Supplementary Material). Relatively potent neutralization was detected against MN.3 and MW965.26 viruses with the neutralization titers for the NFL group lower than the SOSIP group after two immunizations and each subsequent immunization. Against the MW965.26 pseudovirus, the neutralization titers for the NFL group were significantly lower than the SOSIP group (*p* = 0.0355 at P4, *p* = 0.0387 at P5), indicating less V3 exposure, and enhanced *in vivo* stability of the NFL versus the SOSIP trimeric platforms. Relatively weaker neutralization was observed against SF162, HXBc2, and ZM109 pseudoviruses. Against SF162 and ZM109 pseudoviruses, the titers for the NFL group were again lower than the titers for the SOSIP group. In particular, against the SF162 pseudovirus, the neutralization titers for the NFL group were significantly lower than the SOSIP group (*p* = 0.0049 at P4, *p* = 0.0116 at P5). This may indicate why Crotty et al. detected tier 1 neutralization in mice with BG505 SOSIP, whereas we did not (34, 49).

## Discussion

The challenge of protein expression in mammalian cells during the cGMP is to achieve a high-level of expression while also maintaining proper folding and secretion of the “target protein.” Significant advances were recently made in the stabilization and successful production at a research laboratory scale of well-ordered HIV-1 Env trimers from multiple clades (12, 13). The new generation of trimers includes the original SOSIP design, the improved cleavage-independent NFL trimer variants, along with UFOs (10, 12, 50) that are considered native-like in structure. However, subtle variations in quaternary conformations may exist between trimers expressed on the virion surface and the well-ordered soluble trimer variants (51–54). We present here the development of a research grade CHO-M cell line that expresses well-folded BG505 NFL trimers to high homogeneity and yield. We show identification of the best performing pool (pool Z) from transfected CHO-M cell lines, followed by subcloning from pool Z resulting in identification of the well-producing subclone Zcp37. We demonstrate that a second transfection of Zcp37 improves the yield to ~400 mg/l of trimers and that co-transfection of Zcp37ST pool with proprietary auxiliary proteins generate a set of three lead clones Zcp37STcp05-07 that produce ~1.8 g/l of the BG505 NFL trimers at day 9.

We purified the trimers from the cell supernatant from pool Z using a two-step protocol of lectin-affinity and SEC. The trimers produced from CHO-M cell lines were superior to the trimers produced from 293F cell lines in key aspects. (1) Lower fractions of aggregates and aberrant oligomeric forms are generated from the CHO-M cells and (2) negligible binding of CD4 and V3-specific non-bNAbs F105, 19b, B4e8, and 447-52D is detectable to the ordered trimer fraction. These favorable properties may be attributed to the folding mechanisms present in CHO-M cells that reduce aberrant disulfide bond formation and non-specific trimer aggregation. When the trimer peak is analyzed by negative stain EM, it is homogenous with ~90% well-folded trimers and ~10% non-native trimers and displays a favorable antigenic profile with negligible binding of F105 and the non-broadly neutralizing V3-directed antibodies.

Thermal stability analysis by DSC also revealed a slight increase in the melting temperature with the trimers produced in CHO-M cell lines melting at higher T_m_. The minor increase in thermo stability may be due to less variation in the glycosylation profiles of BG505 NFL trimers produced in CHO-M cells. One glycosylation site that is different is at residue N197 where Man_8_GlcNAc_2_ is the most abundant structure on the trimer derived from CHO-M cells and Man_9_GlcNAc_2_ on trimer from 293F cells (21). In addition, the glycan at N637 position is more complex in the trimer produced in CHO-M cells. A comparison of glycosylation profiles of BG505 NFL and BG505 SOSIP both expressed from CHO cells, but purified differently, was also performed (35). Glycosylation differed at two sites: (1) the glycan at N160 position is mixed/complex on BG505 SOSIP, whereas it is exclusively oligomannose on NFL and (2) the glycan at N197 has a higher oligomannose content in BG505 NFL than on BG505 SOSIP.

BG505 NFL trimers derived from CHO-M cell lines elicit strong autologous neutralizing antibodies in rabbits after three subcutaneous inoculations in ISCOMATRIX adjuvant. The neutralization titers against BG505 T332N pseudovirus are potent and uniform with equivalent titers against the BG505 wild-type pseudovirus, indicating that the N332 glycan is not involved in elicitation or detection of neutralizing activity. The N-glycan holes at positions 241 and 289 are the target for neutralizing Abs elicited in rabbits and NHPs immunized with BG505 SOSIP (25, 36, 55). Consistent with those results, all the six rabbits immunized with BG505 NFL also elicit antibodies that recognized glycan holes at one of 230, 241, and 289 positions. This is an important result as it demonstrates that the NFL variant presents the trimers in a native-like conformation to the immune system like the SOSIP trimers, in agreement with our previous comparisons in guinea pigs (29). In fact, in that study, the NFL autologous neutralization titers were higher than SOSIP, but not significantly so. Similarly, in NHPs, BG505 NFL trimers also elicit tier 2 neutralization (25). In this case, the titers are lower than those elicited by SOSIP, but not statistically significantly different. This illustrates immunogenic similarity, with some fluctuations due to either responder animal differences in outbred populations (NHPs: there were a wide range of titers in these animals) or other confounding factors in the complex steps that occur during an *in vivo* biological response. In addition to autologous neutralizations, weak neutralization was also achieved against few heterologous tier 2 viruses from an in-house panel. Development of neutralization breadth has been recently achieved by our group using sequential immunizations with various clades of trimers and glycan masking (31). These results hint that immunizations with a homogenous and stable trimer from a single clade are probably insufficient to develop any breadth against tier 2 viruses and development of a vaccine should include variations in sequence and N-glycan composition.

Elicitation of antibodies against non-neutralizing epitopes on Env may be a distraction to focusing the immune response toward conserved and broadly neutralizing epitopes (23). Antibodies elicited against non-broadly neutralizing epitopes comprising the V3 region typically neutralize tier 1 viruses like SF162, MN.3, and MW965.26, but fail to neutralize tier 2 viruses. Numerous strategies such as glycan masking, engineering additional disulfide bonds, and introducing stabilizing hydrophobic residues in the V3-loop have been employed to reduce V3 exposure *in vivo* (22, 27, 28, 56). As expected, the masking/stabilization of V3 by different strategies has resulted favorably in the reduction of neutralization of tier 1 pseudoviruses. However, in any of these studies, no correlation is detectable between a reduction in tier 1 virus neutralization to improved autologous tier 2 neutralization, or others (28, 57). Nevertheless, eliciting antibodies against non-neutralizing epitopes remains a concern as it might be reflective of trimer “unraveling” during the *in vivo* processes following inoculation (in adjuvant) that increase temperature, involve transport to draining LN, antigen exchange and presentation by FDCs, and proteolytic Env trimer cleavage for presentation for T-cell help, etc. Here, we report that serum from rabbits immunized with CHO-M derived BG505 NFL trimers elicit very robust tier 2 titers, while at the same time elicit weak neutralization of tier 1 viruses. The tier 1 neutralization titers detected here are comparable to the titers achieved in experiments with stabilized versions of BG505 SOSIP and 327c (27, 28, 56). Of note, the BG505 NFL construct does not include mutations to stabilize or mask the V3-loop and yet elicits low levels of tier 1 neutralization. This favorable result (lack of robust tier 1 neutralizing responses) is probably due to the improved homogeneity of the trimers expressed in the CHO-M cell lines that do not expose the V3 region following purification and, more importantly, following multiple rounds of immunization with adjuvant *in vivo*. In support of this conclusion, BG505 NFL trimers from CHO-M cells purified by lectin-affinity chromatography are not recognized by the V3-specific antibodies, 447-52D, 19b, and F425-B4e8 and that five cycles of rapid freeze thaw or storage for 2 months at 4°C do not expose V3 region (Figure S1 in Supplementary Material).

Here, we also compared responses in rabbits to BG505 NFL and BG505 SOSIP expressed in 293F cells with Adjuplex adjuvant. Both the constructs had a C-terminal His-tag that was not removed and were purified in a similar fashion by negative selection. Following five immunizations, serum from rabbits from both the groups displayed equivalent (not significantly different) neutralization titers against BG505 T332N and BG505 wild-type pseudoviruses. These data confirm that the NFL and SOSIP trimers generate similar immune responses as previously reported (25, 29).

**Figure 7:**
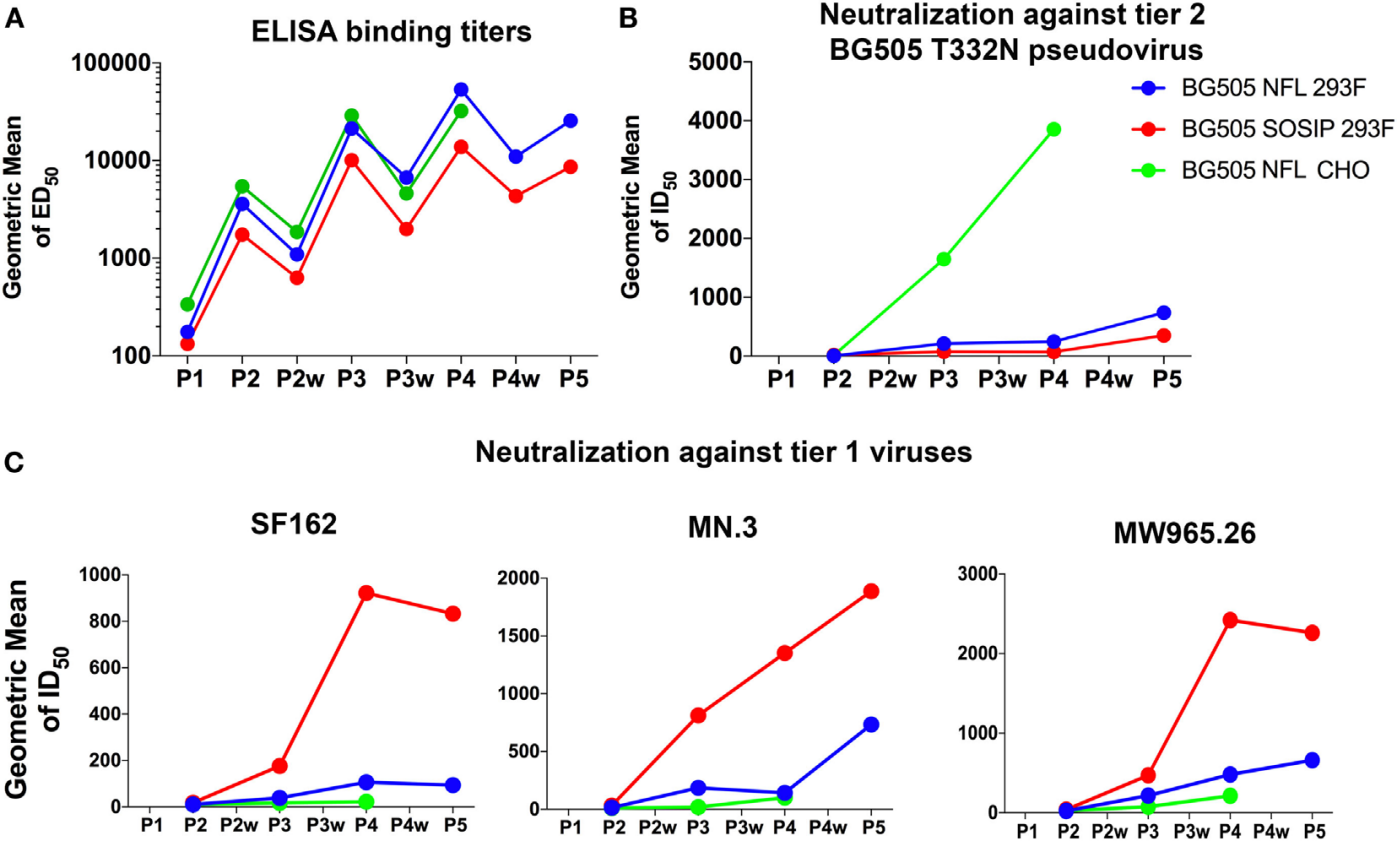
Effect of adjuvant on elicitation of tier 1 and tier 2 neutralizing antibodies. **(A)** Comparison of ELISA binding titers in serum from rabbits immunized with BG505 trimers in ISCOMATRIX [BG505 NFL from CHO-M cells] and Adjuplex (BG505 NFL and BG505 SOSIP from 293F cells) adjuvants after the second, third, fourth, and fifth inoculations. The binding titers are statistically equivalent at all the time points tested. **(B,C)** Comparison of geometric mean of neutralization ID_50_ titers against tier 2 BG505 T332N and tier 1 SF162, MN, and MW965.26 pseudoviruses at the same bleed points. Relatively higher tier 2 and lower tier 1 neutralization titers were observed with ISCOMATRIX adjuvant than Adjuplex indicating that the quality of immune response depends on the effect of adjuvant on the quaternary structure of the trimers and on the subsequent modulation of immune response.

It is interesting to note that all the rabbits immunized with either BG505 NFL or BG505 SOSIP in either ISCOMATRIX or Adjuplex adjuvant elicit similar levels of BG505 NFL- or BG505 SOSIP-specific ELISA binding titers (Figure 7). However, not all the antibodies neutralized antigen-matched tier 2 pseudovirus. The lower level of autologous neutralization elicited with the NFLs in Adjuplex contrasts with the neutralization elicited by the CHO-M-BG505 NFL material, where robust neutralization is detected in all rabbits following just three immunizations (Figure 7), consistent with previous studies using the BG505 SOSIP trimers (22, 23, 25, 27, 33, 36). Little overall structural differences exist between BG505 NFL expressed in CHO-M and 293F cells. The differences in neutralization may be due to improved homogeneity of the trimers produced in the CHO-M cell lines, but more likely is due to adjuvant. We have previously demonstrated that BG505 NFL/SOSIP trimers retained the quaternary structure more effectively in ISCOMATRIX and for longer duration for up to 7 days than in Adjuplex (29). Here, we also demonstrate that the V3 region of BG505 NFL trimers is not exposed when incubated overnight at room temperature in ISCOMATRIX as it is in Adjuplex (Figure S5 in Supplementary Material). Decreased autologous neutralization titers in Adjuplex show that adjuvant-induced conformational changes can affect the display of neutralizing epitopes on the trimers, altering the elicitation of tier 2 autologous neutralization, and increasing tier 1 neutralizing titers (Figure 7; Figure S4 in Supplementary Material).

In summary, we demonstrate the efficacy of Selexis *SUREtechnology*™ platform in developing a research grade CHO-M cell line that expresses native-like, homogenous BG505 NFL trimers at high yields. CHO-M cell lines that efficiently express NFL trimers derived from other clades are currently under development. High homogeneity, low V3 exposure, and low F105 binding of the CHO-M-derived BG505 NFL trimers contributed to elicitation of robust tier 2 and low tier 1 neutralization when formulated in ISCOMATRIX and inoculated into rabbits. Both BG505 NFL and SOSIP trimers elicit tier 2 neutralizing antibodies that are lower in magnitude when formulated in Adjuplex, indicating that non-denaturing adjuvant may better focus the immune response to cross-conserved neutralizing determinants.

## Ethics Statement

The first rabbit study was carried out under subcontract at Covance (Denver, PA, USA), a site approved by the Association for Assessment and Accreditation of Laboratory Animal Care (AAALAC). The Covance Institutional Animal Care and Use Committee (IACUC) approved the study protocol (#0081–16), which was designed and conducted in strict accordance with the recommendations of the NIH Guide for the Care and Use of Laboratory Animals and the Animal Welfare Act and under the principles of the 3Rs. The second rabbit study was carried out at ProSci Inc. (Poway, CA, USA) and is regularly inspected by the USDA (license number 93-R-283) and is OLAW/NIH compliant (Animal Welfare Assurance number A4182-01). All protocols offered are IACUC approved and designed to minimize animal discomfort.

## Author Contributions

SB, AM, VLF, A-JB, RW, NdV, and JLT performed and analyzed the experiments; SB and RTW designed research studies; SB, AM, P-AG, MC, AW, and RTW wrote the paper. All authors reviewed the results and approved the final version of the manuscript.

## Conflict of Interest Statement

The authors declare that the research was conducted in the absence of any commercial or financial relationships that could be construed as a potential conflict of interest.

## Supplementary Material

Figure S1 Characterization and stability of BG505 NFL trimers expressed in CHO-M cell lines. We characterized trimers expressed during the final round of subcloning for structure, antigenic profile, and thermal stability. **(A)** Negative stain electron microscopy; **(B)** ELISA binding analysis; and **(C)** melting profile of trimers by differential scanning calorimetry are shown. **(D)** ELISA binding analysis of BG505 NFL trimers from CHO-M cell lines with selected bNAbs and V3-specific antibodies. Purified trimers were immediately coated on the ELISA plates as a positive control (left). The trimers were subjected to a rapid freeze thaw cycle (5×) (middle) and stored for 2 months at 4°C (right) prior to coating the plates.

Figure S2 Heterologous neutralization responses elicited by BG505 NFL trimers in rabbits. Serum from rabbits immunized with BG505 NFL derived from CHO-M cells weakly neutralized heterologous tier 2 viruses from an “in-house” panel. Neutralization ID_50_ titers for each animal after four inoculations against selected tier 2 pseudoviruses are shown.

Figure S3 Comparison of neutralizing responses elicited by BG505 NFL and BG505 SOSIP trimers in rabbits. Neutralization ID_50_ titers at bleeds points P2, P3, P4, and P5 against a panel of tier 1 and autologous tier 2 viruses as determined by the TZM-bl pseudovirus entry assay are shown.

Figure S4 Comparison of neutralization responses elicited by BG505 NFL and BG505 SOSIP trimers against tier 1 pseudoviruses. BG505 NFL and BG505 SOSIP trimers from 293F cell lines elicited strong responses against tier 1 pseudoviruses. Neutralization titers (ID_50_) against SF162, MN.3, MW965.26, and HXBc2 pseudoviruses elicited by NFL- (blue) and SOSIP- (red) immunized animals are plotted following the third (P3), fourth (P4), and fifth (P5) inoculation.

Figure S5 Stability of V3 region of BG505 NFL trimers in two adjuvants. ELISA binding analysis of BG505 NFL trimers isolated from 293F cells following overnight incubation with **(A)** ISCOMATRIX (75 U of adjuvant with 30 µg of trimer) and **(B)** 10% (v/v) Adjuplex. The V3 region of BG505 NFL trimers is partially exposed in Adjuplex as demonstrated by detectable binding of 19b and F425-B4e8. An increased binding of F105 is also observed with Adjuplex while recognition by F105 is minimal when the trimers were incubated with ISCOMATRIX adjuvant.

Table S1 Library of glycan structures identified on BG505 NFL trimers. Structures were identified and verified by ion mobility mass spectrometry and are represented using the Oxford glycan nomenclature as previously described (58). Da, Dalton; Calc, calculated. Ions are explained below the table.

Table S2 N-linked glycopeptide compositions of trypsin- and chymotrypsin-digested BG505 NFL trimers produced in CHO-M cells identified by liquid chromatography–electrospray ionization-tandem mass spectrometry. Site, N-glycosylation site; XIC, extracted ion chromatogram; Exp., experimental determined mass (shown as a range when different charge states and/or different scans were recorded); Calc., calculated mass. All cysteines are carbamidomethylated. Lower case letters in sequence indicate the position of the modification. Table contains data from two analytical replicates per digest.
