# Supplementary figures and images for: Cleavage-Independent HIV-1 Trimers From CHO Cell Lines Elicit Robust Autologous Tier 2 Neutralizing Antibodies
Source: Front Immunol. 2018 May 24;9:1116. doi: 10.3389/fimmu.2018.01116 (PMC5976746; doi:10.3389/fimmu.2018.01116)

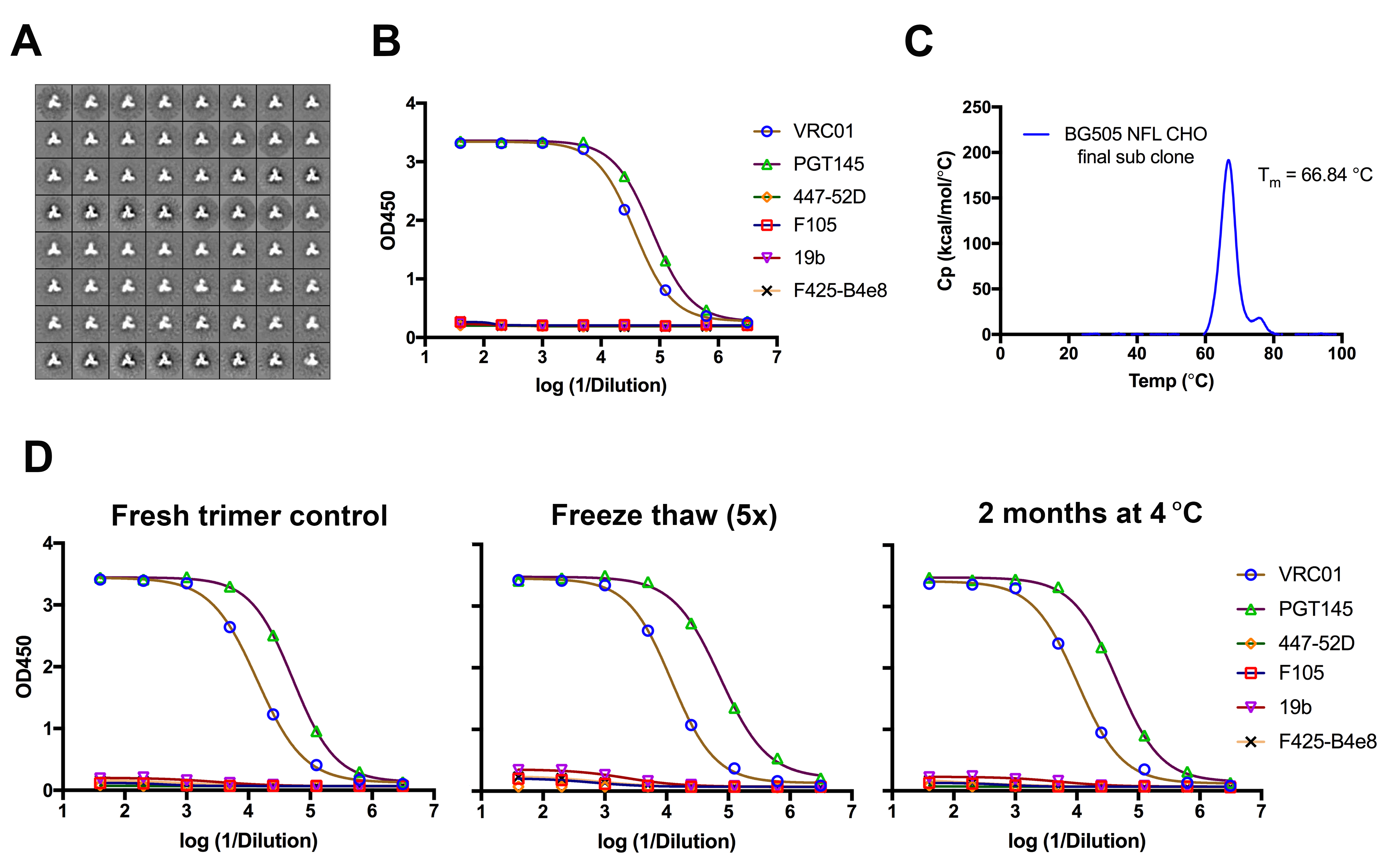

Supplement: Figure S1 — Characterization and stability of BG505 NFL trimers expressed in CHO-M cell lines. We characterized trimers expressed during the final round of subcloning for structure, antigenic profile, and thermal stability. (A) Negative stain electron microscopy; (B) ELISA binding analysis; and (C) melting profile of trimers by differential scanning calorimetry are shown. (D) ELISA binding analysis of BG505 NFL trimers from CHO-M cell lines with selected bNAbs and V3-specific antibodies. Purified trimers were immediately coated on the ELISA plates as a positive control (left). The trimers were subjected to a rapid freeze thaw cycle (5×) (middle) and stored for 2 months at 4°C (right) prior to coating the plates. [file image_1.tif]

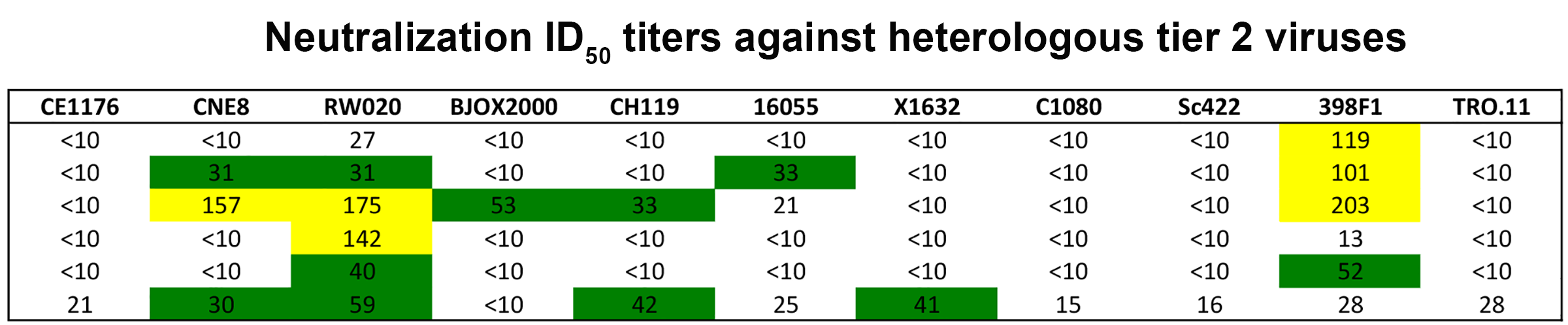

Supplement: Figure S2 — Heterologous neutralization responses elicited by BG505 NFL trimers in rabbits. Serum from rabbits immunized with BG505 NFL derived from CHO-M cells weakly neutralized heterologous tier 2 viruses from an “in-house” panel. Neutralization ID50 titers for each animal after four inoculations against selected tier 2 pseudoviruses are shown. [file image_2.tif]

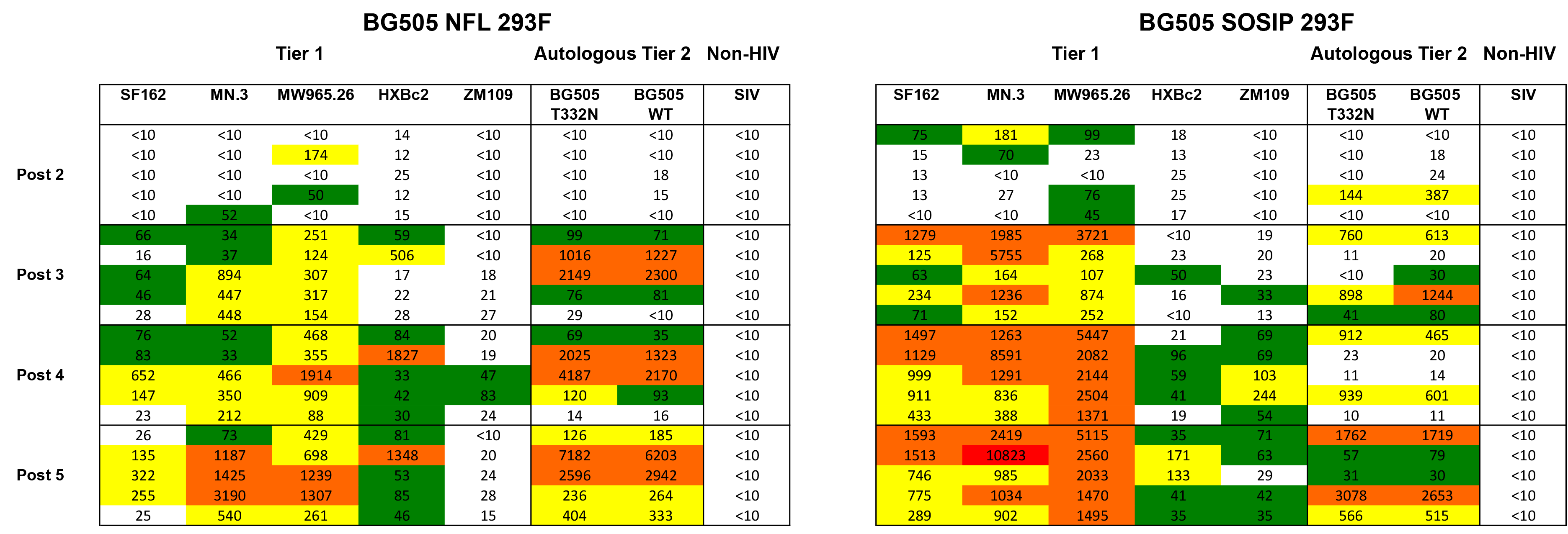

Supplement: Figure S3 — Comparison of neutralizing responses elicited by BG505 NFL and BG505 SOSIP trimers in rabbits. Neutralization ID50 titers at bleeds points P2, P3, P4, and P5 against a panel of tier 1 and autologous tier 2 viruses as determined by the TZM-bl pseudovirus entry assay are shown. [file image_3.tif]

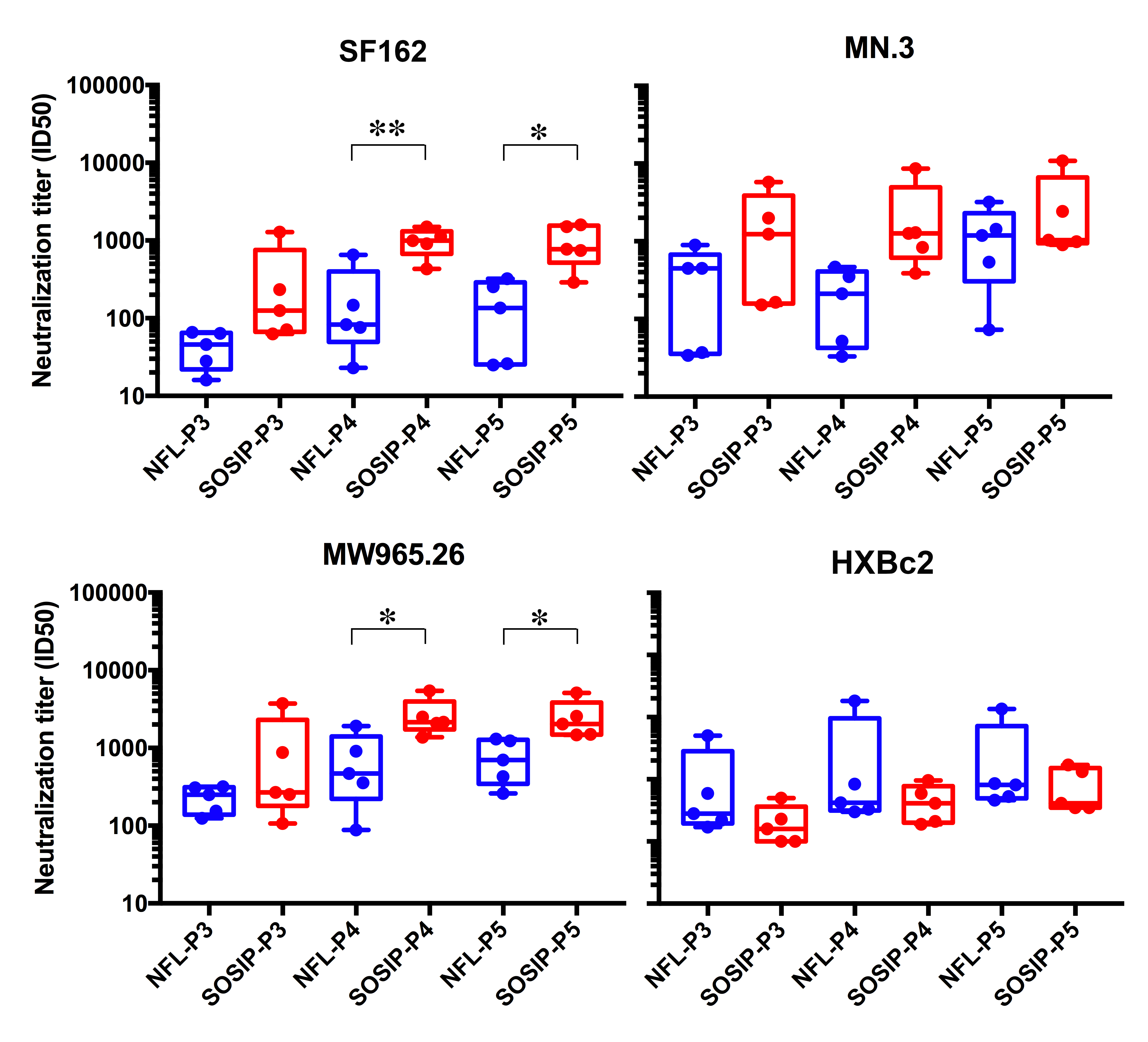

Supplement: Figure S4 — Comparison of neutralization responses elicited by BG505 NFL and BG505 SOSIP trimers against tier 1 pseudoviruses. BG505 NFL and BG505 SOSIP trimers from 293F cell lines elicited strong responses against tier 1 pseudoviruses. Neutralization titers (ID50) against SF162, MN.3, MW965.26, and HXBc2 pseudoviruses elicited by NFL- (blue) and SOSIP- (red) immunized animals are plotted following the third (P3), fourth (P4), and fifth (P5) inoculation. [file image_4.tif]

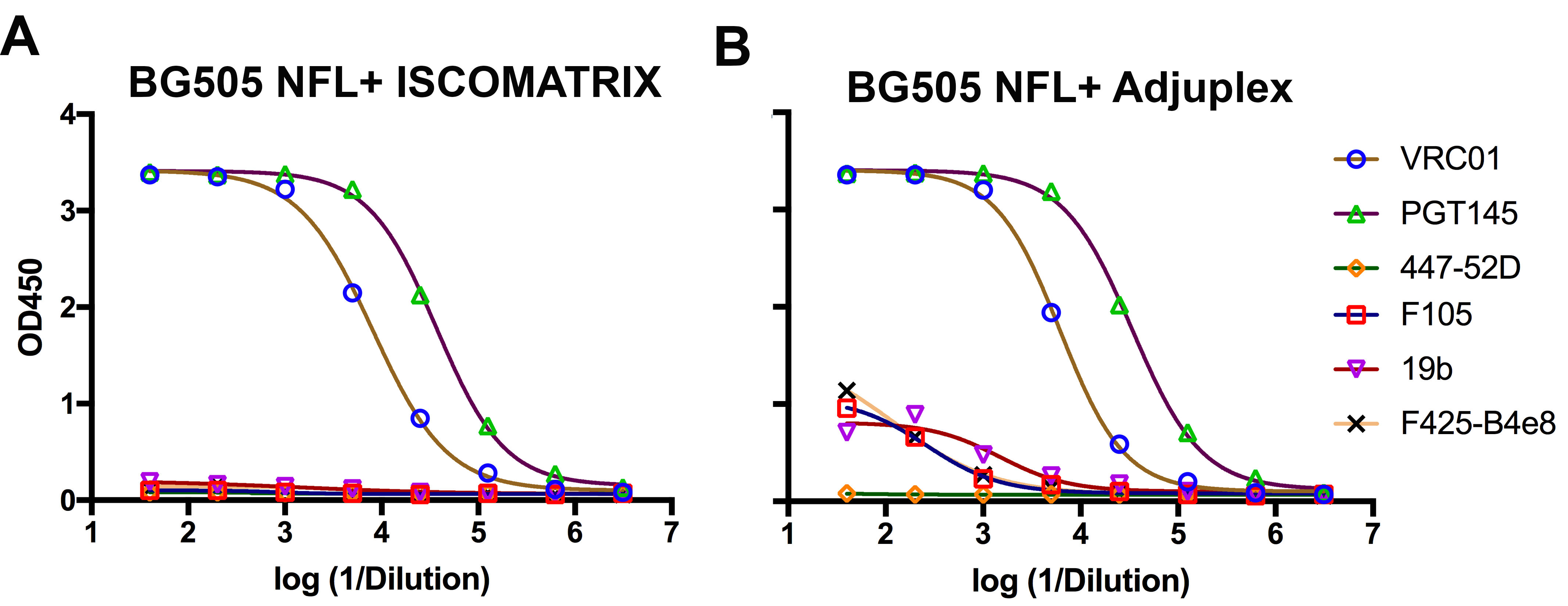

Supplement: Figure S5 — Stability of V3 region of BG505 NFL trimers in two adjuvants. ELISA binding analysis of BG505 NFL trimers isolated from 293F cells following overnight incubation with (A) ISCOMATRIX (75 U of adjuvant with 30 µg of trimer) and (B) 10% (v/v) Adjuplex. The V3 region of BG505 NFL trimers is partially exposed in Adjuplex as demonstrated by detectable binding of 19b and F425-B4e8. An increased binding of F105 is also observed with Adjuplex while recognition by F105 is minimal when the trimers were incubated with ISCOMATRIX adjuvant. [file image_5.tif]
